# Supplementary figures and images for: The endoplasmic reticulum-associated mRNA-binding proteins ERBP1 and ERBP2 interact in bloodstream-form Trypanosoma brucei
Source: PeerJ. 2020 Feb 14;8:e8388. doi: 10.7717/peerj.8388 (PMC7025706; doi:10.7717/peerj.8388)

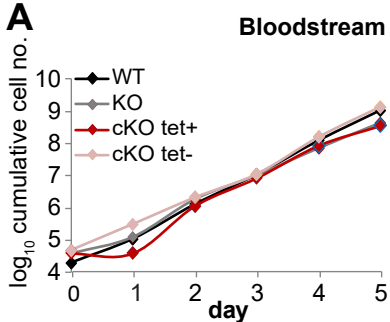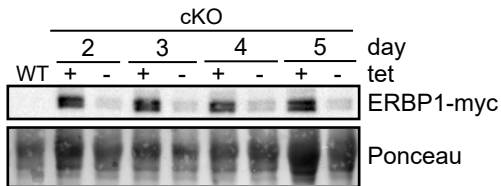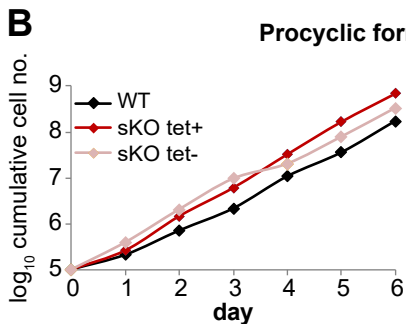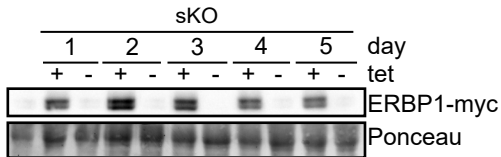

Supplement: Figure S2 — A. Bloodstream-form cells were starved in PBS for 2h then returned to normal medium. Growth is on the left. The lines are: wild-type (WT), knock-out (KO) and conditional KO trypanosomes complemented with tetracycline-inducible ERBP1-myc (cKO) with (+) or without (-) tetracycline. A Western blot showing expression of ERBP1-myc is on the right. B. As (A), except that procyclic forms were starved for 3h. These experiments were done only once. [file peerj-08-8388-s002.pdf]

**A**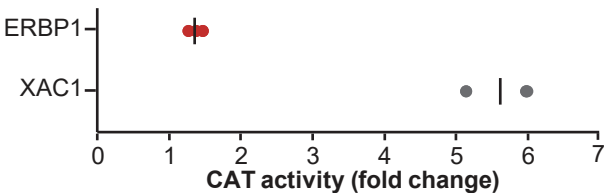**B**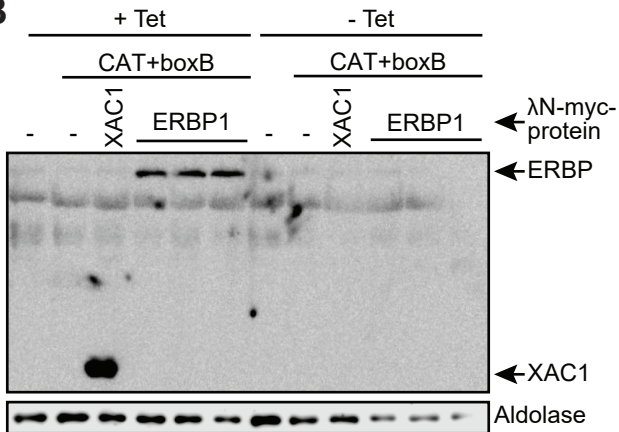

Supplement: Figure S3 — A. Expression of chloramphenicol acetyltransferase (CAT) was measured in cells expressing different lambdaN-myc fusion proteins (ERBP1 and XAC1). XAC1 is Tb927.7.2780, which is a known activator of gene expression, served as control. Expression of the lambda-N proteins was induced with tetracycline (+Tet) for 24h. Results show arithmetic mean (black bar) & individual values of 3 independent experiments. B. Expression of the myc-lambda-N-fusion proteins was validated by Western Blotting. [file peerj-08-8388-s003.pdf]
